# Environmental factors influencing the development of elite athletes: a systematic review

**DOI:** 10.3389/fpsyg.2026.1849040

**Published:** 2026-05-26

**Authors:** Jie Zhao, Xiaowei Peng, Changqing Xiang

**Affiliations:** 1 School of Physical Education, Wuhan Sports University, Wuhan, China; 2 School of Physical Education, Hubei University of Arts and Science, Xiangyang, China

**Keywords:** athlete growth, elite athletes, influencing factors, relationship, talent development

## Abstract

Elite athletes play a vital role in achieving success in competitive sports and have been a significant focus for researchers and coaches alike. To improve the development of elite athletes, this study systematically reviews the environmental factors that influence their growth. This study conducted a thorough search of the Scopus, Web of Science, PubMed, and SportDiscus databases, screening the literature in accordance with the Preferred Reporting Items for Systematic Reviews and Meta-Analyses (PRISMA) guidelines. Ultimately, we identified 15 studies that met our inclusion criteria. The results categorize the influencing factors into four main areas: human resources, family dynamics, team dynamics, and the broader social environment. Notably, the quality of coaching has a considerable impact on the effectiveness of training for elite athletes. The methods of communication used by coaches greatly influence how athletes acquire and develop their skills. Parental support and understanding are also crucial, as they provide the emotional encouragement that elite athletes need. A positive family atmosphere surrounding sports can effectively motivate children to participate in athletic activities. Additionally, a strong bond among teammates creates a supportive team environment, helping elite athletes face challenges with increased confidence. Furthermore, socio-economic factors and social policies significantly affect the training experiences of elite athletes. This study enhances our understanding of the key characteristics that influence the development of elite athletes and emphasizes the essential environmental factors that support their growth.

## 1 Introduction

The significance of elite athletes for the success of competitive sports and the sustainable development of the sports industry is self-evident. They represent a key outcome of effective talent cultivation in competitive sports. In today’s environment, where there is a strong emphasis on developing reserve talent, the training and exploration of elite athletes are gaining increasing attention (Swann et al., 2015; Thomas et al., 2019; McAuley et al., 2021). Especially in some major sports countries such as China, the United States, the United Kingdom, Germany, and Australia, it has always been a key topic of research in the field of sports. In physiology and psychology, the characteristics of human physical and mental development are sequential, phased, unbalanced and individual differences (Lally, 2019). However, individuals have various factors that affect their growth at different stages of development. The development of elite athletes is not an overnight process, it involves a series of selections and professional training. Additionally, various factors influence their progression toward becoming elite competitors (Zhao et al., 2024a). Before exploring these influencing factors, it is essential to gain a comprehensive understanding of what constitutes an elite athlete. According to the Oxford Dictionary of Sport Science and Medicine (3rd edition), elite athletes are defined as those who have reached the highest level in a particular sport (Kent, 2006). In the psychology dictionary of the American Psychological Association (APA), athletes who can compete at the national, international, or professional level are often considered elite athletes (American Psychological Association, 2018). The study indicate that elite athletes are those competing at the international level who can showcase their physical advantages in major competitions, such as the Olympics, World Cup, or World Championships. These athletes establish their elite status through their performance in these significant sporting events (Zhao et al., 2024b). McKay et al. (2022) classified participants into six levels based on their background, athletic ability, and corresponding criteria. The elite level was defined as having the qualifications to participate in international sports events, ranking in the top 4–300 in the world. Research indicates that the definition of “elite athlete” can vary across different sports. For this systematic review, we established a clear and consistent operational definition to determine study inclusion. An athlete is considered “elite” if they meet at least one of the following criteria: (a) they have competed at a national or international level (such as national championships, World Cups, Olympic Games, or equivalent professional leagues); (b) they are members of a national team or a professional sports club; (c) they hold a national or international ranking within the top tier of their sport; or (d) they receive government or institutional funding designated for the development of elite athletes. This definition aligns with the inclusion criteria of this review, which requires that included studies explicitly examine environmental factors (e.g., human, financial, and material resources) affecting the development of such athletes. Studies focusing solely on recreational, collegiate (non-elite), or youth developmental athletes without demonstrated elite status were excluded. The definition was applied consistently by both reviewers during title/abstract and full-text screening.

Numerous studies have examined the factors that either promote or hinder the cultivation and development of elite athletes (Mthombeni et al., 2024; Holden et al., 2025; Bonal et al., 2020; Mallett and Hanrahan, 2004; Sotiriadou and Shilbury, 2009; Sum et al., 2017). These studies can be categorized into three levels of analysis: macro, meso, and micro (De Bosscher et al., 2006). Macro-level research focuses on the social and cultural contexts in which individuals live, taking into account aspects such as population, politics, economic and cultural systems, geography, and climate change. Mid-level research encompasses factors and strategies influenced by sports policies and activities. At this level, the allocation of sports policies, resources, and program was carefully evaluated, and strategies can impact the long-term performance of athletes. Micro-level research may lead to successful elite development, including factors such as individual athletes (such as genetic qualities) and their intimate environment (such as coaches, friends, parents) (Sotiriadou and Shilbury, 2009). Similarly, based on the framework of social ecology, Sum et al. (2017) investigated how social ecological determinants affect the experience of elite student athletes in Hong Kong and Taiwan, China, in the socialization process of dual career development. The results show that elite student athletes are influenced by interrelated determinants at different levels, mainly including the following aspects: (1) personal (career goals, identity, role, personality, self-efficacy, and motivation); (2) Micro level (coaches, teammates, parents, brothers and sisters, PE teachers), other teachers, alumni, seniors, classmates); (3) Middle level (the relationship between individuals and micro level); (4) External factors (government, finance, policies, academia, healthcare, parent teacher associations); (5) Macro level (attitudes, norms, values, beliefs, resources, and culture) and time dimension (transitions). Therefore, this study conducts a specific examination and analysis of the environmental factors that affect the cultivation of elite athletes, to scientifically guide the selection and cultivation of athlete talents.

The purpose of this systematic review was to explore the environmental factors that affect the development of elite athletes from multiple perspectives and disciplines, in order to gain a clear understanding of the relationship between various factors and their influence mechanisms. It aims to summarize their growth and development experiences, understand the patterns in their development, effectively reduce talent turnover rates, and provide coaches with more scientific instructions. Additionally, by clarifying the subordinate factors involved in each type of environmental factor and effectively summarizing these subdivided elements, we can minimize the constraints posed by these factors and nurture more elite athletes. More specifically, this study aims to classify the environmental factors that influence the training of elite athletes. This classification allows coaches, scouts, and stakeholders to gain a clear understanding of the different types of environmental factors, enabling them to develop training plans that are both comprehensive and scientifically grounded. Furthermore, future research could focus on addressing the challenges presented by these environmental factors.

## 2 Methods

### 2.1 Search strategy

This study adhered to the PRISMA 2020 guidelines for conducting systematic reviews and meta-analyses, thereby ensuring methodological rigor and reproducibility. A thorough and predefined search strategy was formulated, integrating pertinent MeSH terms: “Athletes”[MeSH] AND “Environment”[MeSH] AND “Influence Factors”[MeSH]. The detailed search strategy implemented in PubMed is delineated in Table 1. For other databases, including Scopus, Web of Science, and SportDiscus, the core concepts and Boolean operators were adjusted to conform to the unique syntax, field codes, and search protocols of each database (e.g., TITLE-ABS-KEY for Scopus and TS = for Web of Science). The search was confined to literature published up to November 1, 2025, and all retrieved records were exported to a reference management software (EndNote 20) for subsequent screening.

**Table 1:** Search strategy for PubMed.

Search	Query	Results
#4	Search: (((((((((((((((Athletes) OR (Athlete)) OR (Professional Athletes)) OR (Athlete, Professional)) OR (Athletes, Professional)) OR (Professional Athlete)) OR (Elite Athletes)) OR (Athlete, Elite)) OR (Athletes, Elite)) OR (Elite Athlete)) OR (College Athletes)) OR (Athlete, College)) OR (Athletes, College)) OR (College Athlete)) AND ((((((Environment) OR (Environments)) OR (Impacts, Environmental)) OR (Environmental Impact)) OR (Environmental Impacts)) OR (Impact, Environmental))) AND ((((influence factor) OR (influencing factor)) OR (influence factors)) OR (influencing factors))	3,498
#3	Search: (((influence factor) OR (influencing factor)) OR (influence factors)) OR (influencing factors)	889,145
#2	Search: (((((Environment) OR (Environments)) OR (Impacts, Environmental)) OR (Environmental Impact)) OR (Environmental Impacts)) OR (Impact, Environmental)	3,156,747
#1	Search: (((((((((((((Athletes) OR (Athlete)) OR (Professional Athletes)) OR (Athlete, Professional)) OR (Athletes, Professional)) OR (Professional Athlete)) OR (Elite Athletes)) OR (Athlete, Elite)) OR (Athletes, Elite)) OR (Elite Athlete)) OR (College Athletes)) OR (Athlete, College)) OR (Athletes, College)) OR (College Athlete)	466,148

### 2.2 Inclusion and exclusion of studies

According to the purpose and requirements of this study, the following criteria for inclusion in this study are proposed, mainly including three aspects: (1) the language of published articles must be English to ensure the consistency of evaluation; (2) The type of papers is articles, excluding conference papers and book chapters, and they are peer reviewed and the full text is available; (3) The content needs to include all kinds of external or internal environmental factors that affect the cultivation of elite athletes, which can be any aspect of people, money and materials. We excluded those who (1) had nothing to do with elite athletes; (2) did not involve the impact of any element of the training or cultivating environment on elite athletes.

### 2.3 Extraction of data and study eligibility

**Figure 1:**
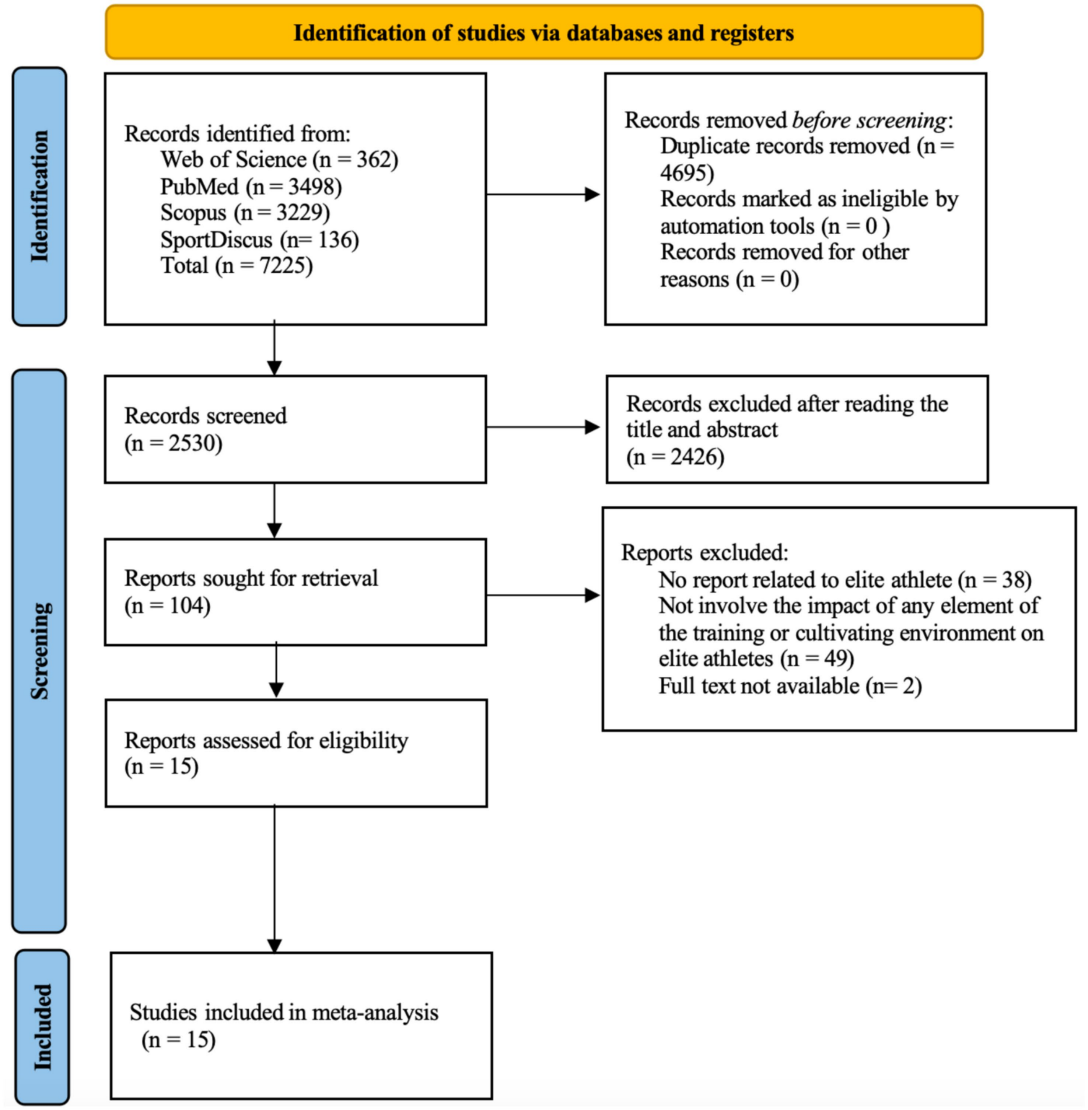
PRISMA flow diagram.

The two authors used a consistent filtering strategy for data extraction and literature screening. After reviewing the titles and abstracts, they eliminated 2,426 articles that were clearly irrelevant to the research topic. A thorough review of the full texts led to the removal of an additional 89 articles, which did not meet the study’s criteria, particularly concerning environmental factors that were either unrelated to elite athletes or not beneficial for their development. The authors, JZ and CX, were responsible for selecting the literature and verifying the included works before finalizing the selection of 15 articles for analysis. Any discrepancies were discussed and resolved, or referred to a third researcher for judgment. The final results were reviewed and approved by all authors. Meanwhile, the 15 selected studies will undergo further quality assessment to determine whether they will be ultimately included. The specific screening process is illustrated in Figure 1.

### 2.4 Quality assessment

The quality assessment of the 15 included studies was assessed using the Joanna Briggs Institute (JBI) Critical Appraisal Checklist for cross-sectional, cohort, and qualitative studies, as appropriate to each study design (Aromataris et al., 2015). The checklist comprises 11 items, each rated as “Yes,” “No,” “Unclear,” or “Not applicable.” The first author (JZ) performed the initial appraisal for all studies. A second author (CX) independently appraised a randomly selected subsample of six studies (40%) to assess inter-rater reliability; any disagreements were resolved through discussion. The detailed results from the JBI assessment of each of the 15 studies are as follows: 11 studies received a “Yes” on 9 or more out of the 11 items, indicating high methodological quality. Three studies scored “Yes” on 7 to 8 items, reflecting moderate quality. One study scored “Yes” on 6 items, primarily due to a lack of clarity in reporting confounding factors and incomplete follow-up data. After evaluation, none of the studies were excluded based on quality; instead, all were given a final decision to be included.

All studies were included, but the quality appraisal directly guided the synthesis in several ways. First, findings from high-quality studies (≥9 Yes) were emphasized more in the overall synthesis of environmental factors influencing elite athlete development. Second, we need to consider sensitivity. One study, which had lower quality (6 Yes), was flagged for potential bias; therefore, its results are interpreted with caution and discussed separately in the synthesis where relevant. Third, we assess the strength of the evidence. The appraisal results are used to inform the strength of evidence statements in the Discussion section (e.g., moderate confidence due to several studies not controlling for confounding variables).

## 3 Results

A total of 15 articles met the inclusion criteria for this systematic review (Table 2 for extracted contents). This relatively small number may be due to: (1) the highly specific focus on environmental factors in elite athlete development; (2) strict inclusion criteria (only empirical, peer-reviewed studies); and (3) the field of elite athlete environmental research is still emerging, and many relevant studies have focused on performance outcomes rather than developmental trajectories, which further limited the eligible pool. Nevertheless, the possibility of missing relevant literature cannot be ruled out, such as the exclusion of grey literature and non-English articles, and database/keyword limitations. Among these, longitudinal research methods and cross-sectional research were employed, with qualitative interviews being the primary research method. In addition, the time period of literature publication is mainly between 2007 and 2025, and the sports involved are the mainstream events of the Olympic Games or the Winter Olympics. Some studies did not mention specific sports, but from the whole Olympic Games. The research reviewed in this study primarily features authors and institutions from European and American countries, with a notable presence from Canada and France. Meanwhile, as a major sports country, China also pays great attention to the research of elite athletes, indicating that European and American countries and China attach great importance to the cultivation of elite athletes. The inclusion of diverse study designs precludes statistical pooling and introduces method-driven heterogeneity. To address this, we conducted a narrative synthesis guided by the methodological framework established by Reed et al. (2021). For each outcome, we first organized the studies by design type. Then, we compared the findings within each design group for consistency before carefully examining patterns across different groups.

**Table 2:** Literature information extraction.

Author/Year	Country	Sports item	Sample	Research design	Key point	Finding
Valverde et al. (2025)	France	Olympic sport representing 32 sports	198 elite French athletes aged 18–49	Cross-sectional study and completion of a series of questionnaires	Socio-environmental conditions (e.g., social environment, sporting status and training organisation, socio-professional situation, and psychological support) contribute to elite athlete career development and related to their motivation.	Socio-environmental conditions also contribute to understanding the maladaptive profile of registered elite athlete and professional athlete. Both adaptive profiles were associated with protective factors that nourish mental health, and the maladaptive profile was linked to more risk factors malnourishing mental health.
Stephan and Brewer (2007)	France	Canoe, wrestling, archery, fencing, rowing, and synchronized swimming	10 retired Olympic athletes, six male and four female participants (mean age = 29.88, SD = 2.82 years)	In-depth, open-ended, and semi-structured interview	Social factors dimension contained four higher-order themes (Socioprofessional flexibility, the coach and the sport staff, peers and teammates as well as social recognition and reinforcement) representing influences from specific environments in elite athlete development.	The present study provides insight into the factors that are potentially distressful for the self-definition and development of elite athletes and could contribute to transitional difficulties.
Dehghansai et al., 2020	Canada	Para sport	Elite parasport athlete	Newell’s constraint-led model	Environmental constraints, including four sub-categories: natural, infrastructure, sociocultural, and interpersonal, were factors that influence elite athlete development and performance.	Environmental factors constraints are less stable (i.e., more dynamic) influences that do not change the goal of the skill and/or sport specific task, but can influence development and performance.
Şenel et al. (2025)	Turkey	Judo	323 national and international Turkish Judo athletes (female = 144, male = 176, and 3 option no to disclose their gender	Questionnaire through Google Drive forms	The coach-athlete relationship is especially important because it provides a conduit that connects the coach and the athlete. Meanwhile, coaches have multifaceted responsibilities that extend beyond just technical aspects of coaching, including supporting athlete development and safeguarding their overall health and well-being, emphasising coaches’ critical role in ensuring psychological safety within the sporting environment.	Good quality relationships where there is trust, respect, commitment and cooperation promote communication and the appropriate support needed, filling athletes with confidence that conversations concerning more sensitive, private and personal matters can occur without fear of negative consequences.
Sauvé et al. (2022)	Canada	Alpine skiing, biathlon, cross country skiing, figure skating, and luge; (basketball, cycling, rowing, soccer, triathlon, and volleyball	Twelve recently retired Canadian Olympic athletes from a variety of winter and summer sports (M_age_ = 33.42, SD = 2.78)	Interviews recorded by Skype (nine interviews), telephone (two interviews), and in-person (one interview)	Three higher order (Interpersonal dynamics, Organizational factors and Intraindividual factors) and nine lower order themes (Coach-athlete relationship, athlete’s support team, athlete’s training environment, finances, planning, communication, result focused mindset, identification with elite sport norms and feelings of isolation versus connectivity) involved in supporting or thwarting Olympic athletes’ well-being.	Elite athletes desire input into the long-term planning and communication of decisions that affect their experience in sport. Also, recurrent success in elite sport is dependent not only on athletic talent but also on effective relationships within an athlete’s sporting environment.
Zhang and Wei (2025)	China	Basketball, table tennis, and athletics	176 athlete in total, 114 were male athletes (64.8%) and 62 female athletes (35.2%); 48 were elite (27.3%) and 128 were sub-elite (72.7%) athletes	Questionnaire (Talent Development Environment, five-point Likert-scale)	Long-term development fundamentals had a significant positive effect on sport commitment for elite athletes, but no significant effect on sport commitment for sub-elite athletes.	Long-term development fundamentals had a positive effect on sport commitment and was only observed in athletes at the elite level.
Arnold et al. (2015)	United Kingdom	Not specific	Fourteen NPDs of Olympic sports (nine male, five female)	Qualitative method (semi-structured interviews)	Environment-related factors consisted of three higher-order themes: development opportunities, operations, and personnel influence NPDS performance, especially the support of family, friends, and partners was highly regarded by many of the NPDs.	The evolution of elite level sport has transformed performance success at an Olympic Games into a multifaceted endeavor that no longer relies solely on an athlete, but also on how effectively his or her entourage deliver their own roles
Solomons et al. (2025)	South African	Rugby	28 women players (mean age: 24.8 ± 4.0 years)	Qualitative research approach (Interviews)	Both coaches and players contribute to creating a positive environment. Additionally, emphasising a coach’s role in encouraging personal growth and fostering a positive sports attitude in athletes.	Coach-athlete relationship is important in cultivating a positive environment for holistic development and sportswoman ship.
Paoletti et al. (2023)	Italy	Judo, artistic swimming and rowing	Six Olympic level female athletes	Qualitative research approach (semi-structured interviews)	Training environment including three higher themes, positive training environment, negative training environment and the impact that COVID-19 had on elite athlete well-being and environment. Also, staff relationship (e.g., coach and manager), teammate’s relationship (including the athletes’ representative), support for the well-being.	Both training, organizational environments and relationships have beneficial elements and negative ones on the Olympic athletes’ well-being.
Dithurbide et al. (2025)	Canada	Olympic or Paralympic sport	25 athletes (17 female, 8 male) from current Canadian national team athletes of an Olympic or Paralympic sport.	Qualitative method (virtual individual interview)	The social and environmental factors that hindered athletes’ mental health included isolation, stressors outside of sport, strained family relationships, and communication fatigue. Meanwhile, a social and environmental factor deemed facilitative by athletes was social support. Coach-athlete relationship and support staff also influence athlete development and well-being.	These social support networks and social environment were imperative for athletes; a concept that was reiterated by mental performance consultants who described social support as one of the main facilitating factors of an athlete’s ability to cope during the pandemic
Georgiades et al. (2017)	Cyprus	Not specific	Elite athlete	Critical review	Training environment (deliberate practice) and environmental factors are undoubtedly both critical to sporting excellence, and the contribution of each is absolutely necessary in the making of a world-class athlete.	A more complete understanding of the interplay between the molecular basis of elite human performance and the environment will also require deciphering the epigenetic response to environmental stimuli.
Franck and Stambulova (2020)	Sweden	Football and basketball	2 team sport senior athlete	Quantitative longitudinal study and interview	Athlete development was influenced by the family environment (e.g., family members, family support). In addition, athletes being motivated mainly by enjoyment of playing sport and social aspects of it (e.g., team spirit, support of key people).	The athletes’ junior-to-senior transition pathway as a multidimensional and multi-factor process that is constructed by athletes through their personal narratives. Therefore, it is important that the sport clubs take the responsibility for creating environments where athletes (despite their ambitions) feel welcomed and have possibilities to develop.
Sarmento et al. (2018)	Portugal	Football	Elite athlete	Systematic review	Athlete development was influenced by the environment constraints, including relative age effect and sociocultural influences. Meanwhile, supportive environments for soccer development seem to have different priorities: (1) social influences and organizational culture during the games and training sessions; and (2) compatibility of the sports practice with familiar, social and school contexts.	Establishing well-integrated youth and senior teams, positive working relationships with parents, and strong and dynamic organizational cultures at elite youth football academies was important for elite athlete development. And the coordination between all staff members in a football academy, such as psychologists, doctors, fitness coaches and directors, also seemed relevant for the players’ personal development.
Xiang et al. (2023)	China	Swimming, badminton, track and field, basketball, and gymnastics	352 elite athletes (M = 13.96 years, 63.35% male)	Quantitative study (five-factor questionnaire and structural equation model)	Training environment, support network, communication with coaches, family characteristics (e.g., parents’ parenting style, family members supportive, socioeconomic status) as well as team conditions (e.g., teammate relationship, team management mode and team experience exchange) were all positive and significant impact on the development environment of sports talent.	Optimizing athletes’ training environment, perfecting the team support network mechanism, improving athletes’ family support for athletes, strengthening effective communication between coaches and athletes, and improving both team management and interpersonal relations will all effectively promote athletes’ development and optimize the wider environment of talent development.
Shelley et al. (2025)	Ireland	Rugby union	92 staff members across all 14 academies	Pragmatic research philosophy method	School and education environment was significant for elite athlete development. And organizational influences considers the organizational factors contributing to, and constraining, academy effectiveness via the progression of players through the different stages in the academy club’s system.	Contextual factors significant impact the efficiency and effectiveness of male academy talent development environments in English rugby union. And complex interactions between a network of individuals and organizations, both internal and external to the structure of the talent system.

### 3.1 Human resource environment

The development of elite athletes is a crucial factor in achieving success in competitive sports. This process reflects the complex decision-making involved for coaches, managers, and scouts as they determine which athletes to include in or exclude from potential talent pools (Baker et al., 2022). As direct stakeholders, coaches exert indisputable influence on training and development and serve as key decision-makers throughout the talent development process (Xiang et al., 2024). Elite athletes are also influenced by their managers within sports organizations. The following sections analyse the human resources environment, focusing on these two roles.

#### 3.1.1 Professional qualities of coaches

While individual talent is significant, a coach with exceptional professional qualities is critical to maximizing athletic potential (Johnson et al., 2011). However, a critical comparison of the available evidence reveals several inconsistencies and gaps that warrant attention. On the one hand, contradictory findings on coach experience versus expertise. Voss et al. (1983) found that expert coaches devote more time to planning and set more precise goals than non-experts. Yet more recent evidence (e.g., Schempp et al., 2010) suggests that experience alone does not guarantee coaching effectiveness; it must be paired with active reflection, learning “on the job,” and adapting to the evolving needs of athletes. The literature lacks a clear, empirically validated threshold distinguishing “expert” from “less expert” coaching in elite development contexts. On the other hand, inconsistent evidence on tactical vs. fundamental focus. Bloom et al. (1999) reported that elite-level coaches prioritise cognitive and tactical elements, whereas beginner/intermediate coaches focus on fundamental principles. But other studies (e.g., Mosher et al., 2022) indicate that tactical emphasis without solid fundamentals may hinder long-term development, particularly in early-specialization sports. This tension remains unresolved: the optimal balance between tactical and fundamental coaching likely varies by sport, age, and competition level, yet no study has systematically tested these interactions.

A harmonious coach-athlete relationship is fundamental to optimal sports performance, effective training, and positive competitive outcomes (Salcinovic et al., 2022; Xiang et al., 2023). As the most essential dynamic in competitive sports (Short and Short, 2005; Şenel et al., 2025), this relationship directly influences success or failure. Effective communication is critical to sustaining this bond (Kim and Park, 2020; Davis et al., 2019), enabling coaches to understand athletes’ needs and challenges (Sauvé et al., 2022), thereby enhancing training performance and long-term development (Davis et al., 2019; Hoffmann, 2025). In turn, athletes better comprehend coaches’ expectations, facilitating efficient execution of training and competition strategies. Both parties thus contribute to a positive environment (Solomons et al., 2025). However, differences in roles, personality, skill levels, and interpersonal complexity create a dynamic and intricate communication structure between coaches and athletes.

#### 3.1.2 Professional competence of managers

Developing elite athletes with well-rounded skills and qualities necessitates strict management systems and principles. A strong athlete management organization can help athletes achieve peak performance and contribute to the long-term growth of competitive sports (Ferguson, 2021). While Ferguson (2021) and Brocherie and Beard (2021) emphasize that managers, athletes, coaches, and other stakeholders must collaborate in talent nurturing, other studies (e.g., Arnold et al., 2015) suggest that managers’ primary functions—talent planning, funding, marketing, and team building—are largely independent of daily coaching. Hence, the literature does not clarify whether management practices directly influence athlete development or operate indirectly through coaching and resource allocation. In another aspect, contradictory evidence on management style and athlete identity. Keshkar et al. (2019) note a complex relationship between sports management and athlete identity, while Stephan and Brewer (2007) suggest that managers’ competition strategies and team models significantly shape athlete trajectories. Yet Kang and Lee (2025) imply that rigid management systems may hinder rather than help elite development, particularly in individual sports where athletes require greater autonomy. This tension—between standardized management processes (e.g., scientific selection criteria, robust systems) and the need for individualized athlete support—remains unresolved. The literature fails to specify when strict management benefits development (e.g., team sports, early stages) and when it becomes detrimental (e.g., elite individual athletes, later stages).

A notable gap in the literature concerns the strength and quality of evidence. Most claims regarding management effectiveness (e.g., Arnold et al., 2015; Ferguson, 2021) are based on expert opinion, case studies, interviews, or non-empirical sources (e.g., position papers, practitioner commentary). Few studies employ quantitative or longitudinal designs to isolate management effects from coaching and environmental confounders. Burns et al. (2022) acknowledge that athlete success requires multiple factors, but the specific contribution of management competence remains unquantified. Accordingly, the evidence base for management factors is weak, dominated by descriptive accounts and theoretical propositions rather than rigorous empirical testing.

### 3.2 Family environment

The family, as a small unit of society, plays a vital role in the growth and education of talent. Family education is the first step toward success for individuals. The family environment is essential for the development of athletes, significantly influencing their talents and abilities (Xiang et al., 2022). In most cases, athletes’ participation in sports will not last long without the support and guidance of their parents (Bonavolontà et al., 2021). And parents’ sports behaviour and patterns have a strong influence on the development of their children’s exercise habits and critical thinking, which will greatly affect their spirit of sports dedication (Huppertz et al., 2017). The lifestyle and values of parents have a direct impact on the development of athletes (Peng, 2022). The family environment plays a crucial role in nurturing and developing sports talent (Taylor and Collins, 2015). A supportive family environment that provides a stable emotional foundation significantly influences the growth of elite athletes.

#### 3.2.1 Parental support

Parental support is indispensable in the development of elite athletes, encompassing motivation, encouragement, and physical, emotional, and financial assistance (Wolfenden and Holt, 2005; Holt and Knight, 2014). Such support promotes youth participation in sports, enhances physical and mental health, and fosters athletic success and passion (Gao et al., 2024). When children face setbacks, parental encouragement helps them persevere (Peng, 2022). Additionally, family understanding provides essential emotional backing, forming the strongest support network for elite athletes (Xiang et al., 2023). Besides, Bloom (1985) and Côté (1999) highlighted that parental support enables elite athletes to sustain specialized practice. Families also make significant financial and time commitments, particularly during the investment years, where parents adopt an advisory role while athletes engage in high-level training and competition. Collectively, parental support is a vital component in elite athlete development.

#### 3.2.2 Family sports atmosphere

In today’s sports culture, there is a strong emphasis on winning, often overshadowing the importance of the process that leads to achieving results. Some families find themselves deeply immersed in this competitive environment and feel a sense of pride, sometimes even arrogance, regarding their children’s accomplishments. However, the development of elite athletes should not be viewed as a way to enhance personal status or achieve personal ambitions. The atmosphere of family sports is related to the enthusiasm of children to participate in professional sports training (Franck and Stambulova, 2020). Parents maintain a high level of interest in their children’s sports and provide emotional support to help them overcome setbacks such as injuries, stress, and fatigue, as well as financial support for training (Baker et al., 2003; Fernanda Porto Maciel et al., 2021). Creating a more interconnected sports atmosphere among family members not only fosters a lasting appreciation for physical exercise but also enhances the family’s enthusiasm for discovering and nurturing sports talents (Franck and Stambulova, 2020). This dynamic can significantly influence the number of individuals pursuing elite sports. Furthermore, John et al. (2019) found in their systematic review that key life events also impact the development pathways of elite athletes. Therefore, from these perspectives, the family’s emphasis on sports and positive attitude towards sports are more conducive to children’s sustained participation in sports (Strandbu et al., 2019), which will also affect their success rate in becoming elite athletes.

More importantly, family sports culture and attitude not only help children develop a lifelong interest and habit of sports, but also form correct sports values. Family sports culture plays a significant role in creating cohesion within families, and the tradition of engaging in sports can greatly influence children’s willingness to participate in sports activities (Badura et al., 2017). For example, many families in Nordic countries have a strong passion for cross-country and alpine skiing. Parents often instill the idea of skiing heroes in their children from an early age, which fosters an environment that supports the development of alpine skiing. This cultural emphasis not only encourages participation but also helps the country nurture and develop elite athletes (Coakley, 2001).

#### 3.2.3 Parenting style

Family is the first environment for personal growth, and the role of family environment and parental upbringing is considered essential for athletes (Yao and Li, 2012). However, a critical comparison of the available evidence reveals some inconsistencies. For example, inconsistent findings on the effects of parenting style. Xiang et al. (2023) report that positive and optimistic parenting styles lead athletes to develop similar attitudes and behaviours. Yet other studies (e.g., Chen et al., 2025) suggest that overly supportive parenting without appropriate challenge may reduce athlete resilience and independence. The literature does not specify the optimal balance between support and autonomy, nor does it distinguish effects by athlete age, developmental stage, or sport type. This gap limits practical application.

Contradictory evidence on family values and participation decisions. Li (2019) attributes the decline in China’s competitive sports reserve talent to changing family values and the family planning policy. However, Yao and Li (2012) argue that the primary barrier is not family values per se, but the perceived academic risk associated with sports training—a finding echoed in the present review’s observation that many families fear negative academic judgments. The contradiction remains unresolved: is the decline driven by structural factors (policy, resource allocation) or cultural factors (stigma, academic pressure)? The impact of this mechanism deserves further empirical exploration. Most claims regarding family influence, such as those by Yao and Li (2012) and Li (2019), are primarily based on cross-sectional surveys or qualitative interviews with small, convenience samples. There is a lack of longitudinal studies that track family dynamics and athlete development over time. Additionally, the evidence supporting the idea that “positive parenting leads to positive athlete outcomes” is largely reliant on self-reports and correlational designs, which makes it impossible to draw causal inferences.

### 3.3 Team environment

Specialized, scientific, and systematic training is essential for developing elite athletes. This training requires appropriate venues, facilities, and a conducive environment as foundational elements. A high-quality team and training environment can provide valuable resources and guidance, facilitating the growth and development of elite athletes. Key factors in this process include the relationships among teammates, the management system, the team’s training approach, and the availability of equipment and facilities.

#### 3.3.1 Teammate relationship

Interpersonal relationships are a key factor in determining the quality of an athlete’s sports experience. Elite athletes do not function in isolation; they are part of a cohesive training team. These relationships encompass a range of connections and interactions that develop between individuals during social interactions (Ye and Ye, 2020). For a country’s team and players, the power of interpersonal relationships is crucial as it can have a significant impact on the team’s development and performance (Burns et al., 2019).

Firstly, good interpersonal relationships and teammates’ relationship can enhance team cohesion and collaboration abilities (Ren, 2009; Paoletti et al., 2023). When teammates build mutual trust, friendships, and harmonious relationships, they are more likely to cooperate effectively, work closely together, and develop an unspoken understanding during games. This phenomenon is particularly evident in team sports such as football, volleyball, and basketball (Salcinovic et al., 2022). The strength of teamwork enhances a sports team’s competitiveness, increases the chances of winning, and supports the development of elite athletes. Secondly, harmonious interpersonal relationships can bring positive attitudes and a positive mental outlook. When teammates support and encourage each other, players become more confident and willing to put in effort for the team. Moreover, with good interpersonal relationships, players are more likely to accept guidance and arrangements from coaches and management, creating a positive team atmosphere. Additionally, strong interpersonal relationships can enhance learning and growth among elite athletes (Burns et al., 2019). When these athletes share and exchange experiences, they can gain valuable insights from one another, leading to continuous improvement in their technical and tactical skills. Interpersonal relationships play a significant role in the psychological well-being and athletic performance of elite athletes (Burns et al., 2022). When there is a strong emotional bond among players and teammates, they tend to be more dedicated and motivated during games, approaching their performance with passion. In other words, an atmosphere of unity, cooperation, and mutual encouragement within a team can boost athletes’ confidence and courage in facing challenges.

#### 3.3.2 Team management and training approaches

The impact of team management and training methods on the cultivation of elite athletes is multifaceted, involving leadership, teamwork, personalized training, psychological development, scientific training methods, and event planning and management, all of which play a crucial role in the growth and development of athletes (Trninić et al., 2009). The sports training management system serves as a guiding principle by offering customized training content. It formulates scientific and well-structured training plans that take into account athletes’ physical conditions, competitive needs, and training objectives. This system helps maintain consistent and stable training, preventing issues related to over-training or under-training. At the same time, the execution of the training plan also requires a long-term tracking and evaluation mechanism to ensure that athletes complete the tasks of each training stage on time, with quality and quantity, and improve training effectiveness and competitive level (Zheng et al., 2020). The training methods used in sports are closely linked to the sustainable development of elite athletes (Baker et al., 2003). Effective team management can help these athletes better navigate their career development paths. It can provide essential training and resources, along with structured career planning and training support. This approach ensures a smooth transition for athletes into other professional fields once they complete their sports careers, thereby offering comprehensive support for their training and development.

### 3.4 Training environment and facilities

The sports training environment, along with sports facilities and equipment, plays a crucial role in athletic activities. The quality and availability of these resources directly influence training and competitive performance (Daniel Tang, 2021). For instance, Paoletti et al. (2023) report that both positive and negative training environments, as well as the COVID-19 pandemic, significantly influenced elite athletes’ well-being and development. Yet the study does not quantify the relative magnitude of positive versus negative effects. Other research (e.g., Henriksen and Stambulova, 2023) suggests that a positive training environment can enhance happiness and performance for elite athletes, but the literature lacks a clear definition of what constitutes a “positive” versus “negative” training environment. Furthermore, no study has directly compared the effects of physical facility quality versus psychosocial training climate; it remains unknown which factor is more influential.

Several studies (Li et al., 2015; Seçkin et al., 2023; Luczak et al., 2019) advocate that wearable devices, artificial intelligence, and performance monitoring systems improve training precision and provide objective data. However, Baker et al. (2003) caution that high-quality equipment may reduce long-distance training costs and enable repeated training, and they indicate that training quality and quantity are two key factors that affect the performance of elite athletes. But they do not address potential downsides—such as over-reliance on technology, data overload for coaches, or reduced athlete intrinsic feedback. The literature contains no systematic evaluation of whether technologically advanced environments produce superior athlete outcomes compared to well-designed low-tech environments. Most claims about training facilities and equipment (e.g., Daniel Tang, 2021; Georgiades et al., 2017; Zhang and Wei, 2025) are based on cross-sectional surveys, expert opinion, or narrative reviews rather than longitudinal or experimental designs. The causal direction is ambiguous: does a high-quality training environment lead to better athlete development, or do more successful athletes receive access to better facilities? No study in this review has used natural experiments or controlled interventions to isolate the effect of facilities from coaching quality, funding, or selection bias.

### 3.5 Social environment

The cultivation of elite athletes, as a distinct educational pursuit, relies not only on specific material conditions, such as the natural and family environments, but also on the broader social and cultural contexts in which these athletes exist. The significant costs associated with the selection and training of elite athletes necessitate substantial funding and support in terms of manpower, facilities, equipment, management, and other resources. Furthermore, the ongoing training and support for elite athletes require long-term stability and commitment. This underscores the importance of the socio-economic development level and social policies, which inevitably impact the nurturing and advancement of elite athletes.

#### 3.5.1 Socio-economic

Socioeconomic factors play a crucial role in enhancing the training levels of elite athletes and are vital for helping them achieve outstanding results (Kelly et al., 2023). The socioeconomic environment directly influences the amount of financial support and resource investment available to elite athletes and serves as the foundation for their development (Xiang et al., 2023). Generally, in regions with favorable economic conditions, governments, businesses, associations, foundations, and other organizations are more willing to invest in cultivating elite athletes. This investment includes providing high-quality training venues, equipment, coaching staff, and other essential resources (Seguí-Urbaneja et al., 2022). Furthermore, in economically developed countries or regions, sports and clubs tend to receive greater attention, and athletes are more likely to gain social recognition and support (Aygün et al., 2023), which is crucial for their growth and development. In another aspect, athlete development and performance was influenced by the social and environment factors constraints, such as socio-cultural and social support (Sarmento et al., 2018; Dithurbide et al., 2025; Dehghansai et al., 2020; Valverde et al., 2025).

Sotiriadou and Shilbury (2009) examined the development process of elite athletes in Australia from an organizational perspective, focusing on 35 national sports organizations (NSOs). Through a document analysis of 74 annual reports from these NSOs over the 4 years leading up to and following the Sydney Olympics, they established a general framework for the development of elite Australian athletes. The study indicates that nurturing successful elite athletes can enhance both the financial aspects and public image of sports, while also fostering greater interest in athletic activities. A qualitative survey examining the factors that influence the development of world-class track and field athletes in the Caribbean revealed three key themes through semi-structured interviews and data transcription: (1) a supportive sports environment, (2) an effective social support network, and (3) important organizational investment. A positive sports environment, combined with strong social and organizational support, can encourage talented Caribbean athletes to pursue and succeed in their track and field careers at a youth level (Thomas et al., 2019). In this context, the socio-economic situation plays a crucial role in shaping elite athletes.

#### 3.5.2 Social policies

Policies play a crucial role in guiding the identification, selection, and development of sports talent. Also, school and education environment was significant for elite athlete development (Shelley et al., 2025). Specifically, they have a direct and significant impact on elite athletes during the talent cultivation process. However, in practice, there is often a lack of coordination between sports policies and educational policies in physical education activities (Fernández-Río and Méndez-Giménez, 2012). Bailey et al. (2009) conducted a national survey in England to assess policies and practices for identifying and supporting talented student athletes in sports. They concluded that a comprehensive school policy is essential because physical education teachers tend to identify talented students based on their current performance rather than their potential achievements. This approach can lead to significant discrepancies in the identification process.

Furthermore, it is crucial to recognize that, in addition to school-level policies, national policies and support from education departments and sports organizations play a pivotal role in nurturing elite athletes (De Bosscher et al., 2011; Zhao et al., 2024a). For example, in China, numerous policies have been established to support the cultivation and development of sports talent in schools, such as the 13th Five-Year Plan for Youth Sports and the Guiding Opinions on Strengthening the Cultivation of Reserve Talents in Competitive Sports. With the guidance of these policies, China’s competitive sports talents have made significant progress in various areas, laying a strong foundation for the development of elite athletes.

## 4 Discussion and conclusions

The present systematic review identified multiple environmental factors influencing elite athlete development, spanning the human, family, team/training, and social domains. Rather than merely cataloguing these factors, this discussion synthesizes findings across the 15 included studies, critically examines their relative influence, and interprets the results through relevant theoretical frameworks. In various studies, the quality of the coach–athlete relationship and parental support have consistently emerged as the two most influential factors. This prominence can be explained using Bronfenbrenner’s (1979) ecological systems theory. According to this theory, both the coach and the family represent the micro-system, which is the immediate environment that athletes interact with on a direct and regular basis. Unlike distal factors such as socio-economic conditions or policies, those within the micro-system have a more immediate, daily, and emotionally charged impact on an athlete’s motivation, self-efficacy, and ability to cope with stress. Several studies in this review (e.g., Davis et al., 2019; Xiang et al., 2023) reported that positive coach-athlete relationships accounted for a larger proportion of variance in training adherence and competition performance than any other single factor. After identifying the factors that influence them, coaches can enhance their qualities in several areas: professional skills, relationships with elite athletes, and communication skills. This improvement will help them better support the growth of elite athletes and achieve outstanding performance. Therefore, coaches should continuously enhance their technical skills through ongoing learning and updates. Moreover, coaches can encourage elite athletes to engage in deep thinking and critically examine their current training situations through positive communication methods. This approach can help athletes overcome emotional blind spots and uncover new possibilities.

Similarly, parental support (Wolfenden and Holt, 2005; Holt and Knight, 2014) was found to be a necessary condition for sustaining long-term specialisation, particularly during adolescence—a developmental period when external motivation is fragile. Consequently, once parents have a clear understanding of the importance of the family’s sporting environment, parental support, and parenting styles in the development of elite athletes, they can effectively adjust their family’s sporting routines, the way they interact with the athlete, and make appropriate changes to their approach to the child’s upbringing. Furthermore, in the family environment, parents should take on the role of “supporters” rather than acting as “coaches” or “managers,” and allow elite athletes the necessary space for independent development. For managers or policy-makers, understanding these influencing factors enables them to recognize their significance more clearly when managing teams and formulating policies, thereby helping to create an environment more conducive to the development of elite athletes. Additionally, managers should respect the values, needs, and individuality of elite athletes. It is important to stimulate their personal motivation and promote a diverse range of management methods, including democratic, emotional, and rational approaches, while avoiding arbitrary control.

In contrast, factors such as training facilities and social policies were identified as enabling rather than driving influences. Their absence clearly hindered development, but their presence did not strongly predict positive outcomes unless accompanied by supportive interpersonal relationships. This pattern suggests a hierarchical structure: socio-material resources form a baseline, while relational factors determine the degree to which athletes translate those resources into sustained development. Rather than treating each factor as independent, the included studies collectively indicate that environmental influences operate through three mechanisms: (a) affective support (emotional encouragement, trust, belonging), primarily provided by parents and coaches; (b) informational guidance (tactical instruction, feedback, career advice), dominated by coaches and senior teammates; and (c) material provisioning (finance, equipment, facilities), involving family and institutional stakeholders. A cross-study pattern emerges: when affective support is high, athletes tolerate moderate deficiencies in material conditions; the reverse does not hold. For example, Lienhart et al. (2020) reported instances where athletes from resource-limited families continued to excel due to exceptional emotional support from their parents. In contrast, several studies indicated that coach-athlete relationships characterized by conflict, despite having access to resources, often resulted in athlete dropout. This finding challenges a purely resource-based perspective on elite development. It aligns with self-determination theory (Deci and Ryan, 1985), which suggests that the three basic psychological needs: autonomy, competence, and relatedness—are mostly fulfilled through interpersonal interactions.

As a result, the main contribution of this study is the identification of environmental factors that influence the development of elite athletes, based on a systematic review of existing literature. This approach differs from previous research, which often focused on isolated and singular influencing factors. The findings reveal that the coach-athlete relationship and parental support are essential, non-substitutable factors in this development, while material resources serve only as enabling resources. However, this study is not without its limitations. One major limitation is that it may not account for certain minor factors among the environmental influences discussed. For example, the relationship between environmental factors and the psychological development of elite athletes—specifically, how the natural, social, and physical environments interact with genetic factors to shape the psychological characteristics of elite athletes has not been fully explored. Additionally, we have not considered the factors that may impact the development of elite sports, such as the natural environment, climate, and geography. On the other hand, this review only focuses on the highly heterogeneous group of elite athletes, and future research can expand to include more adolescent populations. Developing elite athletes is a complex and long-term process that is influenced by various environmental factors. Meanwhile, most of the studies included in this research were conducted at institutions in Europe, North America, and China. For example, in North American countries, the cultivation of elite athletes relies more on social markets, such as professional clubs, sports associations, and other institutions, and the cultivation model is also more commercialized. In China, the cultivation of elite athletes is led by the national government, with a specialized talent selection system, such as the three-tier training network, which completes the selection of athletes based on their competition results and performance, and ultimately trains them in the national team. Additionally, each country has different focuses in its sports programs for elite athletes. For example, Nordic countries often emphasize Alpine skiing and ice sports. Therefore, variations in political systems, cultural backgrounds, training methodologies, and other factors across different countries lead to disparities in the quality of elite athletes’ cultivating. Thus, the findings may not apply to athletes from developing countries or underrepresented regions. Furthermore, future research could explore the advantages that high-income countries have in the development of elite athletes.

## Data Availability

The original contributions presented in the study are included in the article/supplementary material, further inquiries can be directed to the corresponding author.
